# First Sips From the Holy Grail?

**DOI:** 10.1097/TP.0000000000004667

**Published:** 2023-09-25

**Authors:** Tatsuo Kawai

**Affiliations:** 1 Department of Surgery, Massachusetts General Hospital and Harvard Medical School, Boston, MA.

A young kidney transplant recipient came to the emergency room with an elevated creatinine. His biopsy showed evidence of T cell–mediated rejection type 3, and the serum tacrolimus level was undetectable. Despite antirejection therapy, his graft function failed to recover, and the patient returned to dialysis.

Obviously, this is a case of noncompliance with immunosuppressive medications. The usual response in this circumstance is frequently harsh. “The patient should not have received a transplant,” “Another transplant is out of the question,” or “Psychiatric evaluation is warranted.” Assigning blame may seem reasonable in this situation, but is it really fair to put it all on the patient? We take medications to improve our health with the good faith expectation that doing so will “make us feel better or cure what ails us.” However, taking immunosuppressive medication is a double-edged sword. Yes, these chronically prescribed toxins can help prevent graft rejection but not without considerable cost, including countless side effects and unintended complications that significantly increase the risk of death from infection, cardiovascular disease, and cancer.^1^ The COVID-19 pandemic was especially harsh on transplant recipients receiving chronic immunosuppressive therapy, who experienced a mortality rate of 13%–42%^2^ compared with the 1%–2% mortality rate observed in the general population. These observations explain the current 10-y non-death-censored renal allograft survival rate after living and deceased donor kidney transplantation remaining around 65% and 50%, respectively, with 15%–25% of recipients dying despite ongoing graft function.^3^ Faced with these dismal facts, we continue our quest for a more successful alternative—tolerance induction—the Holy Grail of organ transplantation.

In this issue of *Transplantation*, Leventhal et al^4^ report the long-term results of a phase 2 clinical trial for kidney allograft tolerance induction. In this trial, from 3- to 10-y immunosuppression-free kidney allograft survival was achieved in approximately 70% of recipients after persistent chimerism induction accomplished by combining donor hematopoietic stem cell (HSC) transplantation with a conditioning regimen that included the infusion of tolerogenic CD8^+^/TCR^−^ facilitating cells. Immunosuppression was successfully withdrawn in recipients with persistent high-level chimerism (>95% donor cells) but not in those with transient chimerism. Patient and graft survival are comparable with that of recipients receiving ongoing immunosuppression, whereas the estimated glomerular filtration rate was superior in tolerant versus standard of care recipients. Although no comparison has been made with standard of care, no patients in this trial have experienced recurrence of the original cause of their kidney disease (eg, focal segmental glomerulosclerosis and IgA). Encouragingly, chimeric patients were safely vaccinated and developed protective immune responses, and there were no deaths among the 11 recipients with COVID-19 infection.

The apparently required persistent presence of chimerism is considered a reliable biomarker of tolerance, but the authors have provided additional urinary cell mRNA profiles associated with tolerance. It is impressive that the incidence of graft-versus-host disease (GVHD) was low compared with regular bone marrow transplant recipients who received a comparable regimen but without facilitating cells.^5^ Nevertheless, GVHD remains a serious concern in patients with high-level persistent chimerism, and 1 fatal GVHD was also reported in this trial. In more recent phase 3 studies (FREEDOM-1) using the same protocol, a higher incidence of GVHD, including another fatal case, eventually shut down the clinical trial. The more aggressive HSC mobilization strategy in the phase 3 trial may be attributed to the difference in the incidence of GVHD from the phase 2 trial, but safety should be established when using this approach with persistent, high-level donor hematopoietic chimerism.

**FIGURE 1.**
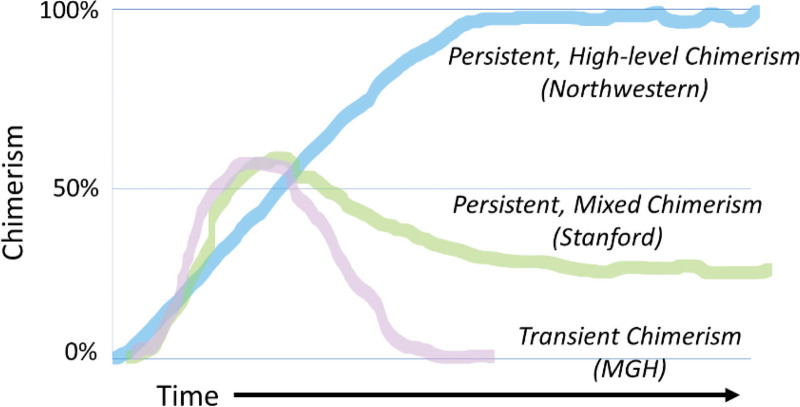
Three chimerism-based strategies for induction of renal allograft tolerance. MGH, Massachusetts General Hospital.

Two other chimerism-based strategies have been employed to induce renal allograft tolerance (Figure 1). Using total lymphoid irradiation, the Stanford group has successfully induced renal allograft tolerance in HLA-matched transplant recipients after inducing persistent or transient mixed chimerism, which is the mixture of host and donor hematopoietic cells.^6^ They carefully adjusted the dose of CD3^+^ cells infused with the HSCs to prevent GVHD while promoting HSC engraftment. However, persistent chimerism has been difficult to achieve with low-level T-cell chimerism in HLA-mismatched transplant recipients, and no allograft tolerance has thus far been achieved in the HLA-mismatched cohort using this strategy.

The Massachusetts General Hospital group has achieved long-term (up to 17 y) immunosuppression-free renal allograft survival in HLA-mismatched transplant recipients after induction of transient mixed chimerism.^7,8^ In this strategy, tolerance appeared to be more dependent on regulatory mechanisms.^9^ Because the chimerism is transient, GVHD is not a concern, but identifying reliable tolerance biomarkers may be critically important because of “presence of chimerism” cannot be used as a biomarker for tolerance.

Seven decades have passed since Billingham et al^10^ published their Nobel-prize-winning report on neonatal tolerance in 1953. As we savor our first taste from the Holy Grail, we do so with the clear understanding that novel immunological interventions will have to be incorporated into the chimerism-based tolerance strategy to make tolerance induction sufficiently easy and safe for universal application.
